# Reticular Formation and Pain: The Past and the Future

**DOI:** 10.3389/fnana.2017.00051

**Published:** 2017-07-05

**Authors:** Isabel Martins, Isaura Tavares

**Affiliations:** ^1^Departamento de Biomedicina, Faculdade de Medicina do PortoPorto, Portugal; ^2^Unidade de Biologia Experimental, Faculdade de Medicina do Porto, Universidade do PortoPorto, Portugal; ^3^Instituto de Biologia Celular e Molecular (IBMC), Universidade do PortoPorto, Portugal; ^4^Instituto de Investigação e Inovação em Saúde, Universidade do Porto (I3S)Porto, Portugal

**Keywords:** serotonin, noradrenaline, analgesics, opioids, emotions, cognition, connectome

## Abstract

The involvement of the reticular formation (RF) in the transmission and modulation of nociceptive information has been extensively studied. The brainstem RF contains several areas which are targeted by spinal cord afferents conveying nociceptive input. The arrival of nociceptive input to the RF may trigger alert reactions which generate a protective/defense reaction to pain. RF neurons located at the medulla oblongata and targeted by ascending nociceptive information are also involved in the control of vital functions that can be affected by pain, namely cardiovascular control. The RF contains centers that belong to the pain modulatory system, namely areas involved in bidirectional balance (decrease or enhancement) of pain responses. It is currently accepted that the imbalance of pain modulation towards pain facilitation accounts for chronic pain. The medullary RF has the peculiarity of harboring areas involved in bidirectional pain control namely by the existence of specific neuronal populations involved in antinociceptive or pronociceptive behavioral responses, namely at the rostroventromedial medulla (RVM) and the caudal ventrolateral medulla (VLM). Furthermore the dorsal reticular nucleus (also known as subnucleus reticularis dorsalis; DRt) may enhance nociceptive responses, through a reverberative circuit established with spinal lamina I neurons and inhibit wide-dynamic range (WDR) neurons of the deep dorsal horn. The components of the triad RVM-VLM-DRt are reciprocally connected and represent a key gateway for top-down pain modulation. The RVM-VLM-DRt triad also represents the neurobiological substrate for the emotional and cognitive modulation of pain, through pathways that involve the periaqueductal gray (PAG)-RVM connection. Collectively, we propose that the RVM-VLM-DRt triad represents a key component of the “dynamic pain connectome” with special features to provide integrated and rapid responses in situations which are life-threatening and involve pain. The new available techniques in neurobiological studies both in animal and human studies are producing new and fascinating data which allow to understand the complex role of the RF in pain modulation and its integration with several body functions and also how the RF accounts for chronic pain.

## Introduction

The involvement of the brainstem reticular formation (RF) in the transmission and modulation of pain is well established. A long path has been covered since the initial anatomical studies demonstrating that the RF projects to the thalamus, passing by the functional approaches showing that manipulation of the RF changes behavioral nociceptive responses and going through imaging studies in humans indicating activation of the RF in response to pain. The study of the role of the RF in pain is challenging due to anatomical and functional reasons. Anatomically, the RF is defined as an aggregation of neurons with several morphological configurations and without distinct connection pattern. Functionally, and besides the sensory component of pain, the RF is involved in a plethora of functions which include arousal, motor reactions, cardiovascular control and visceral functions. This anatomofunctional complexity of the RF deviated neuroscientists from a global study of the involvement of the RF in pain processing and most studies have been directed to specific areas of the RF. Taking into account the concept that pain control cannot be studied apart from other brain functions and also the intrinsic feature of the RF as the brain network, by excellence, it is possible that the RF represents an outstanding example of the “dynamic pain connectome” (Kucyi and Davis, 2015, 2016).

**Figure 1:**
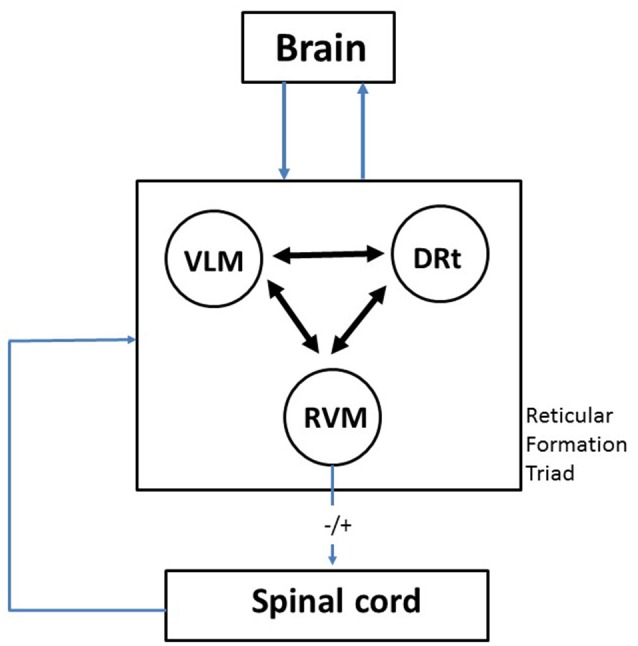
Diagram depicting the crucial role of the medullary reticular formation (RF) triad rostroventromedial medulla (RVM)-ventrolateral medulla (VLM)-dorsal reticular nucleus (DRt) as a gateway between the brain and the spinal cord. The three components of the triumvirate are reciprocally connected in a way which allows integration of responses which are related to pain, namely cardiovascular control and motor reactions. This probably represents a phylogenetically conserved mechanism that subserves the classical “fight or flight” response, activated by acute pain. The emotional and cognitive control exerted by higher brain centers upon the RVM-VLM-DRt triad may account to explain how the emotions and attention affect pain responses. By allowing to balance inhibition and facilitation (−/+) of nociceptive transmission at the spinal cord, the RVM-VLM-DRt medullary triad provides the mechanism to decrease or increase pain. A more detailed description of the neuronal circuits involving one component of the triad—the DRt—is shown in Figure 2, namely in what concerns its brain connections and their neurochemical characterization.

Based on our own experience in the study of the involvement of specific areas of the brainstem RF in pain transmission and modulation in animal models of pain, in this article we critically review the participation of the medullary RF in pain modulation. The medulla oblongata contains three areas of the RF from which a wide variety of data have recently been collected, namely the rostroventromedial medulla (RVM), the caudal ventrolateral medulla (VLM) and the dorsal reticular nucleus (also known as the subnucleus reticularis dorsalis; DRt). After reviewing the anatomofunctional features of the involvement of each area in pain transmission and modulation, we then discuss how the triad RVM-VLM-DRt plays a key role as a gateway to allow pain modulation from the brain to target the spinal cord, i.e., top-down modulation (Figure 1). The role of the RVM-VLM-DRt triad in pain processing is proposed as a homeostatic neuronal circuit that allows adequate body reactions in life-threatening events, such as escaping a predator or an intense acute pain (McNaughton and Corr, 2004; Mobbs et al., 2007). Additionally the triad RVM-VLM-DRt is also proposed to be involved in situations in which adequate emotional responses to pain are necessary (Craig, 2003).

## Ascending and Descending Pathways

The involvement of the spinoreticulothalamic pathway as a major ascending pathway for nociceptive transmission to the brain is well established. Overall this multisynaptic pathway originated from neurons mainly located in the spinal cord laminae IV–V and VII–VIII targets areas of the medullary and pontine RF which have collaterals of the spinothalamic tract (Lu and Willis, 1999). A role for the RF as a relay to the medial thalamus has emerged and herein conferred the RF an interesting perspective as to its involvement in the motivational-affective components of pain (Willis and Westlund, 1997; Almeida et al., 2004). As to the areas of the RF directly receiving nociceptive information from the spinal cord, our research group performed extensive neuroanatomical tract-tracing studies showing that spinal neurons projecting to the VLM or to the DRt are strongly activated in response to several types of nociceptive stimuli (Tavares et al., 1993; Almeida and Lima, 1997; Castro et al., 2006).

The brainstem RF is not only targeted by nociceptive input from the spinal cord but is also actively involved in modulation of nociceptive transmission from the spinal cord. The association of the RF to descending pain modulation is almost as ancient as the discovery that the periaqueductal gray (PAG) is involved in stimulation-produced analgesia (Heinricher et al., 2009). The PAG plays a critical role in conveying the modulatory influences from higher brain centers involved in aspects of pain responses such as cognition and emotion, including the insular and prefrontal cortices and the amygdala (Tracey and Mantyh, 2007). The PAG collects the modulatory influences from these areas and uses the RVM as a relay to indirectly target the spinal cord. Another contrasting and yet important aspect of the participation of the brainstem RF in descending pain modulation derives from the direct arrival of nociceptive input from the spinal cord, namely to the VLM and DRt, described in the next section. In contrast to the RVM, which is does not receive afferents from the spinal cord, the participation of the VLM and DRt in reciprocal loops established with the spinal cord and better analyzed in the next section, allows the RF to perform a fine tuning of nociceptive signals traveling from the spinal cord to the brain.

## An Important RF Triad in the Medulla Oblongata

In this section we will summarize the anatomical and functional data that support an involvement of the RVM, VLM and DRt in the transmission and modulation of nociceptive information. The specific features of each area will be outlined in each subsection according to the following organization: (i) anatomical location; (ii) connections with the spinal cord; (iii) nociceptive control; and (iv) local neurochemical systems.

### The Rostroventromedial Medulla

#### Anatomical Location

The RVM includes the midline located nucleus raphe-magnus (NRM) and the adjacent RF in the vicinity of the nucleus reticularis gigantocellularis. In what concerns its involvement in pain modulation, these anatomical components of the RVM are frequently considered together inasmuch that local modulation techniques, such as electrical or chemical stimulation, do not allow a precise discrimination of the NRM from the adjacent RF. Rostrocaudally, the RVM extends from the pontomedullary junction to the level of appearance of the pyramidal decussation.

#### Connections with the Spinal Cord

The ascending projections from the spinal cord to the RVM are very scarce. The descending input from the RVM to the spinal cord is prominent and targets almost all spinal segments, reaching mainly the dorsal horn (laminae I–V) but also lamina X (Millan, 2002). As to the way in which RVM fibers are organized in spinal neuronal circuits, it was proposed that the descending input from the RVM directly targets spinal neurons involved in ascending transmission of nociceptive input (Urban and Gebhart, 1999). These data are based in the use of tract-tracing techniques and pharmacological manipulation of the spinal cord circuits. According to recent opto/chemogenetic manipulations of the RVM, another arrangement may exist namely by GABA-mediated inhibition of inhibitory spinal interneurons that presynaptically inhibit primary afferent fibers (Francois et al., 2017). This demonstrates that the new techniques used to manipulate neuronal circuits, namely optogenetic manipulation, will allow to uncover the complexity of top-down modulation.

#### Nociceptive Control

The involvement of the RVM as a crucial relay station from the pain modulatory actions arising from the PAG is well established (Fields et al., 1991). For comprehensive reviews see, for example Heinricher et al. (2009) and Ossipov (2012); for a recent update see Heinricher (2016). In animal studies it was shown that the RVM has a peculiar bidirectional role in pain control at the spinal cord namely by balancing inhibitory (antinociceptive) and facilitatory (pronociceptive) effects at the spinal cord. One of the best established neurobiological mechanism of bidirectional control from the RVM is the existence in the RVM of two classes of neurons. OFF-neurons are involved in pain inhibition and their electrophysiological responses pause when the animal exhibits a nocifensive behavior. On the contrary, ON-neurons are involved in pain facilitation as their electrophysiological activity increases just prior to the appearance of a nociceptive withdrawal reflex. Besides the two types of neurons, the RVM also harbors NEUTRAL neurons which exhibit an electrophysiological activity that is not related to identified animal behavior. The coexistence of antinociceptive and pronociceptive systems at the human RVM was only recently studied. This delay in evaluating the putative translational perspectives of the existence of OFF- and ON- neurons is mainly due to the limitations of imaging techniques in the study of small regions, such as the RVM. Initial imaging studies which managed to evaluate RVM activation in healthy volunteers confirmed activation of this RF region in response to nociceptive stimulation of healthy volunteers (Fairhurst et al., 2007; Eippert et al., 2009). More recently, and by using a brainstem optimized whole brain imaging protocol, it was possible to demonstrate distinct clusters of activity in the RVM of healthy volunteers, which match the antinociceptive and pronociceptive activities of OFF-and ON-neurons, respectively (Brooks et al., 2017). The translational perspectives of this recent imaging study need to be evaluated in the future, namely to analyze if activation of pronocicetive components of the RVM increases in chronic pain patients, in a manner similar to the well-established results in animal models (Carlson et al., 2007; Gonçalves et al., 2007; Silva et al., 2013).

#### Local Neurochemical Systems

The role of serotonin in local control of RVM neurons has been demonstrated. In fact, and besides targeting the spinal cord, serotoninergic RVM neurons have been shown to modulate the activity of RVM neurons (Potrebic et al., 1994; VanderHorst and Ulfhake, 2006). The RVM also contains opioid-sensitive neurons since the activity of OFF-neurons is activated by mu-opioid agonists whereas the opposite occurs with ON-neurons (Heinricher et al., 1992; Heinricher and Fields, 2013). The local neurochemical control appears to be more complex and involve GABA-mediated inhibition which is triggered by opioids. Local cholecystokinin receptors (CCK2) are also relevant and provide additional possibilities of a fine tuning of neurochemical control at the RVM. An additional neurochemical system was recently unraveled at the RVM. The endovanilloid system is remotely activated from the PAG since agonists of the vanilloid receptor type 1 (TRPV1) injected into the PAG induce glutamate-mediated activation of the activity of OFF neurons (Starowicz et al., 2007; Palazzo et al., 2010). The endovanilloid system at the RVM appears to be inactivated in non-noxious conditions or during acute pain (Silva et al., 2016b). The system is only activated at the RVM in situations of chronic pain, namely during neuropathic conditions (see below in “Chronic Pain as a Trigger of Neuroplasticity at the RVM-VLM-DRt Triad” Section).

### The Caudal Ventrolateral Medulla

#### Anatomical Location

The caudal VLM is located in the ventrolateral quadrant of the caudalmost aspect of the medulla oblongata in several species including man, rat, mouse, cat and monkeys. The VLM extends from the spinomedullary junction up to the level of the rostral border of the area postrema. The caudalmost part of the VLM contains an area designated as VLMlat, which is located between the spinal trigeminal nucleus (Sp5C) and the lateral reticular nucleus (LRt) and appears to play a specific role in pain modulation (Tavares and Lima, 2002, 2007). The VLM also includes the LRt, which is a nucleus with a specific pattern of connections and has been known for its involvement in motor responses (Alstermark and Ekerot, 2013).

#### Connections with the Spinal Cord

The ascending projections from the spinal cord to the VLM are anatomically segregated, with the VLMlat receiving mainly afferents from the superficial dorsal horn, namely from noxious-responding neurons located in lamina I (Tavares et al., 1993). An important contingent of VLMlat-projecting neurons is located at lamina II, which appears to be a special feature of this spinofugal pathway (Lima and Coimbra, 1991; Lima et al., 1991). On the contrary, the LRt is targeted from fibers originated from deeper layers in the spinal cord, namely from laminae IV–V, which specifically terminate in the lateral part of the LRt, and from lamina VII which terminate at the medial part of the nucleus (Lima and Coimbra, 1991; Lima et al., 1991).

Our research group performed several anatomofunctional studies to evaluate the areas of termination of descending pathways from the VLM to the spinal cord, taking into account the anatomical segregation reported above as to the spino-VLM pathway. The descending VLMlat-spinal pathway targets lamina I, IV–V and X and appears to course through the dorsolateral funiculus (Tavares and Lima, 1994; Tavares et al., 1996). A reciprocal loop between lamina I and the VLMlat was further characterized at the ultrastructural level (Tavares and Lima, 2002). Terminal boutons from lamina I establish mainly asymmetrical contacts with VLMlat neurons that project to the spinal cord, suggesting that the ascending input from the spinal cord directly activates VLMlat neurons. In the spinal cord, descending projections from the VLMlat establish both asymmetrical and symmetrical synapses (Tavares and Lima, 2002). Collectively the data suggest that the arrival of noxious input from the spinal cord triggers activation of VLMlat neurons. Detailed electrophysiological mapping of the VLM has shown that it harbors inhibitory neurons (OFF-like neurons) along with excitatory cells (ON-like cells; Pinto-Ribeiro et al., 2011) which, along with our ultrastructural data (Tavares and Lima, 2002), indicates that the descending modulation from the VLM may include facilitatory modulation, along with the well-established inhibitory effects.

#### Nociceptive Control

The anatomical data reviewed above are easy to conciliate with functional findings showing that the magnitude and duration of behavioral nociceptive responses is more intense when the VLMlat is stimulated (Gebhart and Ossipov, 1986) in comparison with stimulations directed to more medial VLM areas. The VLMlat contains neurons that respond to noxious activation of the joints (Pinto et al., 2007). Incidentally, it must be noted that the magnitude of activation of VLMlat neurons, as measured by the expression of the c-*fos* protooncogene, is directly correlated with the magnitude of activation of lamina I neurons (Pinto et al., 2006), which reinforces the functional relevance of the above mentioned lamina I-VLMlat-lamina I loop. Recent studies showed that VLMlat neurons respond to diffuse noxious stimulation of the muscles (Panneton et al., 2015) and a new role for the VLMlat as a component of the classical spinoreticulothalamic pathway has emerged in which this RF region is proposed to have a key relay role. The poor activation of more medial components of the VLM, namely the LRt, in response to noxious stimulation along with the role of the LRt in motor control suggests that the VLM may be specially positioned to provide an integrated response to an acute stimulus, namely to the classical “fight or flight” response. In fact, a recent study proposed that the spino-LRt-cerebellar pathway provides an adequate motor response in response to noxious peripheral stimulation (Huma et al., 2015). An additional component of this response involves cardiovascular parameters. The VLMlat is activated in response to increases in blood pressure (Tavares et al., 1997a; Lima et al., 2002). Increases in blood pressure are also a feature of the “fight or flight” response. Collectively, the data suggest that the VLM is an integrative center which is involved in producing the adequate pain, motor and cardiovascular responses necessary to face threatening events.

#### Local Neurochemical Systems

The local neurochemical circuits relevant to control the functions of the VLM in pain modulation appear to involve noradrenaline and angiotensin II. As to the former, administration of noradrenaline or the α_2_-adrenoreceptor agonist clonidine into the VLM inhibit local neurons and produce hyperalgesia (Cahusac and Hill, 1983; Ossipov and Gebhart, 1986). Angiotensin II injected into the VLM also induces hyperalgesia which is mediated by local angiotensin type 1 receptors (AT_1_ receptors; Marques-Lopes et al., 2010). By a selective manipulation of the noradrenergic projections from the pontine A_5_ noradrenergic cell group to the VLMlat, it was proposed that VLMlat neurons expressing AT_1_ receptors activate A_5_ noradrenergic neurons which will inhibit nociceptive transmission at the spinal cord. That triggering action of the VLM is inhibited by collaterals of the descending A_5_-spinal pathway (Tavares et al., 1997b). Another important neurochemical control system at the VLMlat is mediated by opioids. At the VLM, μ-opioid receptors are expressed mainly by VLMlat neurons that do not project to the spinal cord (Pinto et al., 2008b) and overexpression of opioids at the VLM induces antinociceptive effects (decreased behavioral nociceptive responses) and lower nociceptive spinal neuronal activation (Martins et al., 2011).

### The Dorsal Reticular Nucleus

#### Anatomical Location

The DRt is located in the caudal-most aspect of the medulla oblongata in several species including man, rat, cat and monkey. The DRt extends from the spinomedullary junction up to the level of the rostral border of the area postrema. It confines with the cuneate nucleus and the nucleus of the solitary tract (NTS), dorsomedially, the Sp5C, laterally and the VLM, ventrally (Andrezik and Beitz, 1985; Newman, 1985).

#### Connections with the Spinal Cord

**Figure 2:**
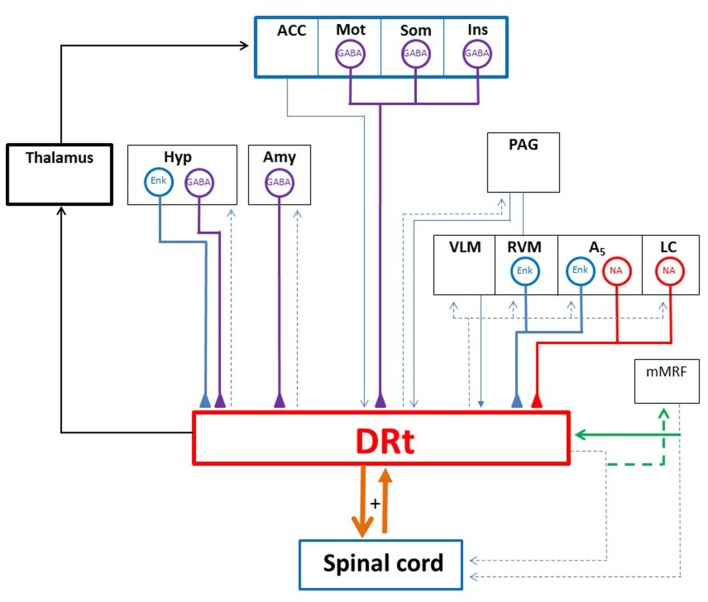
Diagram depicting the DRt connections with the spinal cord and several brain areas. The DRt is involved in a feedback reciprocal loop with the spinal cord (*thick orange lines*) which is involved in pain facilitation. Through its projections to the lateral ventromedial thalamus the DRt participates in a reticulo-thalamo-cortical ascending nociceptive pathway (*thick black lines*). The DRt receives afferent inputs from: (i) higher centers namely the anterior cingulate cortex (ACC), the motor (Mot), somatosensory (Som) and insular (Ins) cortices, the hypothalamus (Hyp) and the amygdala (Amy); and (ii) several brainstem areas namely the periaqueductal gray (PAG), the locus coeruleus (LC), the A_5_ noradrenergic cell group along with the two components of the medullary triad (RVM and VLM). The DRt is a major relay for descending pain facilitatory inputs from the ACC, the Hyp and the noradrenergic LC and A_5_ areas. The neurochemical characterization of DRt afferents showed that afferents originated in the Mot, Som and Ins cortices as well as the Hyp and the Amy are GABAergic (GABA; *thick purple lines*). Hyp afferents and brainstem afferents located at the RVM and the A_5_ area are enkephalinergic (ENK; *thick blue lines*). The LC and the A_5_ noradrenergic cell group constitute the main source of noradrenaline (NA; *thick red lines*) released at the DRt. A peculiar reciprocal network is established between the DRt and the medial medullary RF (mMRF) through collaterals (*green lines*) of spinally descending axons. Such an arrangement was described in the cat and is thought to be involved in noxious sensing and nocifensive behavior. Blue thin lines represent neurochemically uncharacterized DRt afferents and dashed lines represent DRt or mMRF efferents.

In what concerns the connections with the spinal cord, our research group has shown that DRt neurons are reciprocally connected spinal lamina I neurons forming a reverberative nociceptive circuit (Figure 2). The DRt receives bilateral projections from spinal cord laminae I, IV–VII and X with an ipsilateral predominance of those originated in the dorsal horn (Lima, 1990; Villanueva et al., 1991). Spino-DRt pathways travel though the dorsal columns, with the projections originated in the superficial dorsal horn traveling through the dorsal funiculus and those originated in the deep dorsal horn traveling in the dorsolateral fasciculus (Lima, 1990; Almeida et al., 1995; Lima and Almeida, 2002). The lateral aspect of the ventrolateral quadrant also constitute an ascending tract used by spino-DRt pathways, most likely originated from the deep dorsal horn, since lesions including the most lateral parts of the ventral funiculus prevented the activation of DRt neurons, contrary to lesions including the dorsal funiculus where fibers originated from superficial dorsal horn neurons run (Bing et al., 1990). DRt neurons project to the superficial and deep dorsal horn (Bernard et al., 1990; Tavares and Lima, 1994; Villanueva et al., 1995). DRt-spinal pathways reach the spinal cord through the dorsolateral funiculi (Villanueva et al., 1995).

The termination of DRt neurons in the same laminae containing spinal neurons projecting to the DRt predicts the existence of reciprocal circuits between the spinal cord and the DRt. A reciprocal excitatory loop between the DRt and superficial dorsal horn neurons was demonstrated. At the superficial dorsal horn, excitatory contacts, demonstrated at the ultrastructural level, occur between DRt descending fibers impinging upon lamina I neurons, which project back to the DRt. At the DRt, excitatory contacts were also demonstrated between fibers ascending from spinal lamina I neurons and local neurons (Almeida et al., 1993, 2000). Lamina I neurons projecting to the DRt convey nociceptive inputs to the DRt as they express c-Fos, a marker of spinal neuronal nociceptive activation (Hunt et al., 1987), upon noxious stimulation (Almeida and Lima, 1997). This reciprocal excitatory loop likely forms a reverberating circuitry feeding both the spinal cord and the DRt with nociceptive information and ultimately amplifying it. The deep dorsal horn and the DRt are also likely linked by reciprocal links as suggested by anatomical and electrophysiological data. The latter showed that activation of the DRt by glutamate injection exerts excitatory influences on deep dorsal horn neurons as observed by the long lasting increase of the responses of wide-dynamic range (WDR) neurons to noxious electrical stimulation of the sciatic nerve (Dugast et al., 2003). On the contrary, blocking the ipsilateral DRt by lidocaine produces an immediate decrease of C-fiber-evoked responses to sciatic nerve stimulation postdischarge activity of WDR neurons (Lima and Almeida, 2002).

#### Nociceptive Control

The DRt has been assigned a peculiar role in pain processing, with most data gathered in the rat and cat. In humans, the recent developments of functional magnetic resonance imaging (fMRI), allowing to investigate more accurately the spinal cord as well as the brainstem, also confirm the involvement of the DRt in pain processing (Rempe et al., 2014, 2015; Youssef et al., 2016). Electrophysiological *in vivo* recordings performed in the rat identified two neuronal subpopulations at the DRt, total nociceptive convergent neurons which are exclusively activated by noxious stimuli conveyed by Aδ- and C-fibers from the entire body, and partial nociceptive convergent neurons, which are activated by both noxious and innocuous stimuli and are targeted by C-fibers innervating only restricted areas of the body and by Aδ-fibers originated in extensive peripheral areas (Villanueva et al., 1988, 1989). In addition to responding to and encoding cutaneous and visceral noxious inputs, DRt neurons responded in a manner proportional to the intensity of stimuli (Villanueva et al., 1989; Roy et al., 1992). These electrophysiological properties were also found in DRt neurons of the monkey (Villanueva et al., 1990) and cat (Soto et al., 2008). Recent electrophysiological *in vivo* and *in vitro* studies performed in the cat and the rat, respectively, showed that a large fraction of DRt neurons presented spontaneous activity (Soto and Canedo, 2011; Sousa et al., 2014). Additionally, DRt neurons show windup (Villanueva et al., 1988), a phenomena that consists of a gradual build-up in neuronal excitability, generated in response to low frequency C-fiber afferent input or generated supraspinally (Soto et al., 2008; Soto and Canedo, 2011). This physiological property of DRt neurons is important inasmuch as wind-up has been interpreted as a system for the amplification of pain which could lead to the development of central sensitization (Herrero et al., 2000).

The DRt facilitates acute and short-lasting inflammatory pain (Almeida et al., 1996, 1999; Ambriz-Tututi et al., 2013; Martins et al., 2013, 2015a). Stimulation of the DRt by local administration of glutamate increased nociceptive acute behavioral responses while DRt lesion by local administration of quinolinic acid had the opposite effect (Almeida et al., 1996). Chemical or electrical lesioning of the DRt decrease inflammatory pain behaviors (Almeida et al., 1999). Spino-DRt-spinal excitatory loops are likely involved in the mediation of the pain facilitatory actions observed after formalin injection since both DRt lesioning or blockade of glutamate receptors at the DRt decreases formalin-induced pain behaviors and c-Fos expression in superficial and deep dorsal horn laminae of the spinal cord (Almeida et al., 1999; Ambriz-Tututi et al., 2013).

The DRt is also involved in diffuse noxious inhibitory control (DNIC), a mechanism of top-down inhibitory control of spinal dorsal horn neurons (Bouhassira et al., 1992b). DNIC is a paradigm in which one noxious stimulus is used as a conditioning stimulus to induce reduction in pain perception by another stimulus. DNIC is triggered exclusively by a conditioning noxious stimulus, conveyed by peripheral Aδ- and C-fibers, and inhibit trigeminal and spinal convergent WDR neurons (Le Bars et al., 1979; Dickenson et al., 1980). DNIC is sustained by a complex loop, involving supraspinal structures, whose ascending and descending pathways travel through the ventrolateral and dorsolateral funiculi, respectively (Villanueva and Le Bars, 1995). Early electrophysiological and lesioning studies established that pathways originated from the DRt are involved in DNIC (Bouhassira et al., 1990, 1992a,b, 1993). It was suggested that descending inhibitory inputs from the DRt constituted a separate type of inhibitory control, and it was hypothesized that DNIC triggered from the DRt could be part of a mechanism involved in the extraction of nociceptive information by depressing background body sensory activity (Le Bars, 2002).

The DNIC inhibitory control also occurs in humans (Yarnitsky, 2010; van Wijk and Veldhuijzen, 2010). More insights into DNIC have been gathered namely that it recruits cortical networks, which may explain why pain expectation, which relies on cortical structures, prevents DNIC (Goffaux et al., 2007). In line with this, a recent fMRI study performed in humans suggest that a lack of DNIC results from increased control of the DRt from the cortex (Youssef et al., 2016). Another interesting finding likely helping to comprehend DNIC modulation, is that DNIC involves the activation of opioid receptors at the DRt (de Resende et al., 2011). Interestingly, our work shows that opioids inhibit DRt descending facilitation, it is therefore likely that the expression of DNIC might also rely on a balance between descending inhibition and facilitation, with notably descending facilitation overpowering descending inhibition. Another evidence that pain facilitation likely opposes the effects of DNIC comes from a study showing that chronic treatment with morphine, which induces paradoxical opioid-induced hyperalgesia (Lee et al., 2011), was shown to eliminate DNIC by activating pain facilitatory neurons in the RVM (Okada-Ogawa et al., 2009). Additionally, several other recent studies show that descending spinal inhibitory noradrenergic pathways are necessary for the expression of DNIC (Bannister et al., 2015; Peters et al., 2015). Finally, DNIC is decreased in patients and animal models with chronic pain and studies using the rat show that pharmacologically increasing noradrenaline, which is deficient at the spinal cord during neuropathic pain (Hughes et al., 2013), as well as blocking serotoninergic descending facilitation mediated by 5-HT3 receptors, restores DNIC in neuropathic animals (Bannister et al., 2015). Taking these recent findings into account and given the fact that chronic pain results from an imbalance between descending inhibition and facilitation with the imbalance towards increasing descending facilitation (Ossipov et al., 2014), assessing DNIC in patients could potentially constitute a relevant tool to monitor alterations in the endogenous pain modulatory pathways (van Wijk and Veldhuijzen, 2010), which could potentially help identify patients at risk for development of chronic pain and ultimately help find better pain treatments.

#### Local Neurochemical Systems

Pain facilitation from the DRt is modulated by several neurotransmitters such as glutamate, opioid peptides, noradrenaline and GABA. The excitatory amino acid glutamate plays a key role in the pronociceptive actions of the DRt during the formalin test since the blockade of AMPA/KA, NMDA and mGlu1 glutamate receptors by the local administration of the respective antagonists significantly reduced formalin-induced pain behavior which was accompanied by a reduction of c-Fos expression at both the superficial and deep dorsal laminae (Ambriz-Tututi et al., 2013). The tonic activity of glutamate at the DRt likely results from the sustained peripheral afferent input, induced by formalin injection, leading to increased activation of spino-DRt-spinal reverberative circuits (Almeida et al., 1993, 2000). Noradrenaline is also involved in the mediation of pronociception from the DRt. Indeed, noradrenaline release at the DRt, measured by *in vivo* microdialysis, increases during the formalin test (Martins et al., 2013). The reduction of noradrenaline release at the DRt by genetic manipulation of DRt-noradrenergic afferents significantly attenuated pain behavior in the formalin test while increasing local extracellular levels of noradrenaline, by inhibiting its recapture, produced the opposite effect (Martins et al., 2013). The genetic manipulation of DRt-noradrenergic afferents was performed by a viral vector derived from the Herpes Simplex virus type 1 (HSV-1) which is retrogradely transported from the DRt to its noradrenergic afferents, namely the locus coeruleus (LC) and A_5_ noradrenergic cell group (Figure 2), where it selectively reduces noradrenaline synthesis (Martins et al., 2010). The pain facilitatory actions of noradrenaline at the DRt are mediated through activation of α_1_-adrenoreceptors (Martins et al., 2013).

Opioid peptides were shown to inhibit DRt pain facilitation as shown by HSV-1-mediated overexpression of enkephalin at the DRt, which produced anti-hyperalgesia in a model of acute pain (Martins et al., 2008). Opioids likely act through direct inhibition of DRt spinally projecting neurons since these neurons express μ-opioid receptors (Pinto et al., 2008a,b). Opioids also act the DRt through additional inhibitory mechanisms, likely by disinhibiting enkephalinergic interneurons which receive input from GABAergic interneurons expressing μ-opioid receptors and being presynaptically inhibited by δ-opioid expressing-fibers (Pinto et al., 2008a). Opioid peptides responsible for the anti-hyperalgesic action found in our studies are mostly released from local interneurons but also from DRt afferent sources namely the RVM, the A_5_ noradrenergic cell group and the hypothalamus (Figure 2; Martins et al., 2008).

The inhibitory neurotransmitter GABA is involved in the mediation of pronociception from the DRt. Our recent studies show that GABA release at the DRt, measured by *in vivo* microdialysis, increases during the formalin test and that it increases DRt pain facilitation through activation of GABA_B_ receptors (Martins et al., 2015a). Indeed, GABA_B_ receptors knock-down at the DRt, mediated by lentiviral vectors, or the pharmacological blockade, via the local administration of a GABA_B_ antagonist, significantly attenuated formalin-induced pain behavior while the local administration of a GABA_B_ agonist induced the opposite (Martins et al., 2015a). The effect of GABA is likely due to disinhibition of the DRt spinally projecting neurons since a large proportion of GABA_B_ receptors are expressed by local opioidergic neurons inhibiting DRt spinally projecting neurons (Pinto et al., 2007, 2008a; Martins et al., 2008). GABA might be released from local interneurons but also from insular, somatosensory and motor cortices (Figure 2) which represent the most important afferent pathways to the DRt and they are GABAergic (Martins et al., 2015a).

## The RVM-VLM-DRt Triad as a Key Gateway for Top-Down Pain Modulation

The three components of the triad of RF medullary addressed in this review (RVM, VLM and DRt) are interconnected (Figure 1). The VLM↔DRt reciprocal connection is likely to have functional implications since the magnitude of nociceptive neuronal activation in two regions in response to nociceptive stimulation is positively correlated (Pinto et al., 2006). Similar correlation studies should be enlarged to the RVM.

The RVM-VLM-DRt triad is in a privileged position to collect input from higher brain centers and convey this modulation to the spinal cord (Figure 1). The RVM conveys input from the PAG which, on its turn, is an area that integrates modulatory influences arising from the areas involved in cognitive and emotional aspects of the pain response, such as the frontal cortex and the amygdala (Tracey and Mantyh, 2007). Furthermore, the RVM-VLM-DRt triad has the unique feature of being involved in bidirectional pain modulation, namely by balancing pain inhibition and pain facilitation (Figure 1). The triad is acted by the main relevant neurotransmitters involved in pain modulation, namely opioids, noradrenaline and serotonin, which has a putative translational value since most pain killers act at the opioidergic and monoaminergic systems. We therefore propose that the triad RVM-VLM-DRt is a key gateway in top-down directional pain modulation (Figure 1).

Due to our expertise in the study of the DRt and its peculiar role in pain facilitation, this part of the triad should be analyzed in detail. The DRt is a major integrative relay for ascending nociceptive information participating in a reticulo-thalamo-cortical pathway (Figure 2) through which the DRt may allow any signal of pain to again access to widespread areas of the neocortex and thus help prime the cortex for attentional reactions and/or the coordination of motor responses (Bernard et al., 1990; Villanueva et al., 1998; Monconduit et al., 1999, 2002; Desbois and Villanueva, 2001). The DRt receives projections from several brain areas predominantly from several cortical areas, the hypothalamus, the amygdala and brainstem areas involved in descending pain modulation (Figure 2). Besides the RVM and VLM, the DRt receives projections from the PAG, and a strong noradrenergic input from the pontine noradrenergic cell groups LC, A_5_ and A_7_ (Almeida et al., 2002).

The DRt is also a major integrative relay for descending nociceptive modulation from several brain areas including the cortex (Zhang et al., 2005), the hypothalamus (Amorim et al., 2015) and the brainstem (Martins et al., 2013; Velo et al., 2013; Leiras et al., 2016). The DRt was shown to relay descending pain facilitation from the anterior cingulate cortex (ACC; Zhang et al., 2005). Additionally, the DRt receives dense innervation from cortical areas, namely from the motor (M1 and M2), somatosensory (S1 and S2) and insular cortex (Desbois et al., 1999; Almeida et al., 2002). Our recent work demonstrated that a high percentage of the cortical projections to the DRt were GABAergic (Figure 2) and the release of GABA at the DRt enhanced DRt pain facilitation (Martins et al., 2015a). Therefore, it is likely that the DRt might relay descending facilitatory actions triggered from these cortical areas. Moreover, given the involvement of some of this cortical areas in the emotional and cognitive control, the GABAergic projections from these areas may represent a neuronal circuit that accounts for pain enhancement during imbalance of emotional and cognitive conditions. The DRt also relays descending nociceptive facilitation induced by galanin in the dorsomedial nucleus of the hypothalamus (Amorim et al., 2015). Our recent functional studies show that the DRt acts as an important relay station in the mediation of descending facilitation from noradrenergic areas, namely the LC and A_5_ noradrenergic groups (Martins et al., 2013). The LC exerts a bidirectional role on pain modulation (Hickey et al., 2014) and its pain facilitatory role is relayed by the DRt (Martins et al., 2010, 2013, 2015b).

The DRt is also involved in a network of reciprocal connections established between collaterals of DRt and medial medullary reticular spinally-projecting neurons (Figure 2; Leiras et al., 2016). Medial medullary reticular neurons are involved in nociceptive and escape motor responses (Casey and Morrow, 1989), it is therefore likely that the DRt might integrate noxious sensing and nocifensive behavior through the collaterals of descending axons with medullary reticulospinal neurons.

The extensive ascending and descending connections of the RVM-VLM-DRt triad allow it to modulate the spinal nociceptive signals and integrate those signals with environmental and emotional conditions providing adequate and adaptable pain responses while preserving the homeostasis of the organism. Based on the phylogenetically conserved nature of the RF, we propose that the RVM-VLM-DRt triad is a conserved part of the brain and represents a vital gateway which evaluates and controls pain responses and triggers adequate and integrated responses. In a very recent review, a role for the brainstem RF in emotional control has been discussed in detail (Venkatraman et al., 2017). The brainstem has been proposed the key region to provide the effective link between sensory and effectors networks in processing emotions. By harboring components of the emotional motor system, the RVM-VLM-DRt triad can provide both the rapid “fight or flight” responses necessary to provide a rapid and specific body reaction but also the responses adequate to process “slow burning” somatic and visceral pain signals (Parvizi and Damasio, 2001; Craig, 2003). Based on the intrinsic interconnections of the RVM-VLM-DRt triad, it may also represent, by excellence, an example of the structural connectome of the central homeostatic network (Edlow et al., 2016), namely in its involvement in pain responses. Pain is a main challenger of the body homeostasis, both in acute pain when cardiovascular and motor reactions should be rapidly adjusted, and in chronic pain, when anxiety, depression and even neurodegeneration can occur.

## Chronic Pain as a Trigger of Neuroplasticity at the RVM-VLM-DRt Triad

The data gathered in several animal models point to a participation of the RVM-VLM-DRt triad in chronic pain. It remains to be ascertained if that RF triad plays a similar role in the human brain but the still low resolution of imaging techniques does not allow detailed studies of those medullary regions. The interesting perspectives derived from recent imaging studies (Brooks et al., 2017) is likely to provide important data in a near future.

During chronic inflammatory pain several studies indicate that the activity of the triad is altered. Increased activity at the VLM and DRt were detected in a functional study based in the consumption of 2-D-glucose in chronic monoarthitic rats (Neto et al., 1999). In the specific case of the DRt, this could be related both to increased spinal excitatory inputs to the DRt in addition to a loss of inhibitory opioidergic tone into the DRt. The former was suggested to occur due to an imbalance between excitatory and inhibitory actions exerted upon spino-DRt neurons. Spinal dorsal horn neurons projecting to the DRt are under direct inhibitory GABAergic modulation, as they express mainly GABA_B_ receptors (Castro et al., 2006) but in the spinal dorsal horn of monoarthritic animals there are lower percentages of GABA_B_ expressing neurons and higher percentages of NK1 neurons (Castro et al., 2005) which are being subjected to excitatory actions exerted by substance P. A loss of inhibitory opioidergic tone likely occurs at the DRt since monoarthritic rats show decreased expression levels of of μ- and δ-opioid receptors at the DRt (Neto et al., 2008; Pinto et al., 2008a). These hypotheses are consistent with the results of a recent study showing the involvement of the DRt in formalin-induced secondary allodynia and hyperalgesia through a tonic glutamatergic excitatory tone (Ambriz-Tututi et al., 2013). Indeed, the administration of glutamate antagonists into the DRt reduced both secondary allodynia and hyperalgesia and their development was prevented by pretreatment with glutamate antagonists injected into the DRt before formalin injection (Ambriz-Tututi et al., 2013). The results of this study further support a key role for glutamate receptors into the DRt in the development of formalin-induced secondary allodynia and hyperalgesia.

Neuropathic pain is associated with spinal neuronal sensitization (Laird and Bennett, 1993), an activity-dependent expression of hypersensitivity of spinal neurons. Electrophysiological studies suggest a contribution of the DRt to the maintenance of spinal sensitization during neuropathic pain (Sotgiu et al., 2008). The recent studies performed by our group implicate the noradrenergic modulation of the DRt in the enhancement of DRt pain facilitation during neuropathic pain. We showed that nociceptive stimulation increased noradrenaline release at the DRt which enhanced pain facilitation from the DRt through activation of α_1_-adrenoreceptors (Martins et al., 2015b). Reducing noradrenaline release at the DRt by using a viral vector derived from the HSV-1 which selectively reduced noradrenaline synthesis in noradrenergic DRt afferents, significantly attenuated the behavioral manifestations of neuropathic pain for nearly 2 months (Martins et al., 2010). Our studies also show an impairment of the feedback inhibitory function of α_2_-adrenoreceptors at the DRt during neuropathic pain, which likely further contributes to enhance the noradrenergic input to the DRt during neuropathic pain (Martins et al., 2015b). The increased noradrenergic neurotransmission at the DRt, enhancing pain facilitation from this area, raises an important issue related to the treatment of neuropathic pain with antidepressants inhibiting noradrenaline reuptake. Noradrenaline reuptake inhibitors are known to produce analgesia through a spinal action but our results also show that they may enhance pain facilitation from the brain counteracting thus their analgesic effects at the spinal cord.

The occurrence of irreversible changes during the installation of chronic pain should also be considered since it was demonstrated that the RVM suffers neurodegeneration during the installation of neuropathy. In initial phases of neuropathy, the RVM exhibits plastic changes in what concerns its involvement in descending modulation namely by increases in the activity of pain facilitatory ON-neurons and opposite for OFF-neurons, along with an enhancement of pain facilitatory actions at the spinal cord mediated by local 5-HT_3_ receptors (Silva et al., 2013, 2016a; Guo et al., 2014). Collectively these changes lead to increase in descending pain facilitation which may account for chronic pain installation. During the progression of pain, and due to the continuous barrage of nociceptive input from the spinal cord, a disruption of local RVM circuits occurs with the appearance of massive oxidative stress damage and hyperactivation of glial cells (Silva et al., 2016a). The subsequent neuroinflammation may lead to neurodegeneration, associated with neuronal loss, which represents a non-plastic effect of chronic pain installation at the RVM. Neuroplastic changes in key descending pain modulatory areas were shown to be crucial for the generation and maintenance of chronic pain (Woolf and Salter, 2000), the studies compiled here strongly suggest that the alterations occurring at the RVM-VLM-DRt triad during chronic pain are also fundamental to its maintenance. Additionally, our results highlight the necessity of taking into account the alterations occurring at the levels of the RF for the design of efficient therapies for the challenging treatment of chronic pain.

## Future Directions in the Study of the Reticular Formation

Functional neuroanatomical studies rely on the coupling of neural tracing with other neuroscientific techniques such as electrophysiology and behavior. In the last few years these studies have been massively propelled by the use of novel technologies such as optogenetics and chemogenetics which allow simultaneously manipulating circuitries and linking those circuits to behavioral outputs. Optogenetics use channels that are activated by light (Copits et al., 2016), while chemogenetics uses engineered G protein-coupled receptors, that are activated by otherwise inert drug-like small molecules, to activate or silence neuronal firing (Roth, 2016). Hitherto, optogenetics have been successfully used to study brain circuits such as those involved in regulation of the sensory and affective aspects of pain, namely cortico-limbic networks (Copits et al., 2016). Chemogenetics have also been successfully used to examine spinal circuits involved in pain modulation (Bourane et al., 2015; Peirs et al., 2015; Saloman et al., 2016) and the study of descending pain modulation is also starting to be approached by both technologies (Cai et al., 2014; Hickey et al., 2014; Jurik et al., 2015; Li et al., 2016). The RF contains areas relaying and integrating nociceptive information, from the brain and the spinal cord with other physiological functions. In the future the use of optogenetics and chemogenetics will allow a more comprehensive and precise study of the integrative role and the specificity of the intricate circuits of the RF.

At the clinical setting, the technical improvements at the brain imaging field will also allow to study the RF in more detail. By proposing that the medullary RF has a triad of pain control centers (the RVM-VLM-DRt), the technical problems of studying small components may be surpassed as the triad should be considered as a gateway from the brain to the spinal cord. The RVM-VLM-DRt triad probably represents an example of the “brain pain connectome” and should not be analyzed as to its individual components, *per se*. Future studies will analyze the RVM-VLM-DRt triad as a phylogenetically conserved structure of the RF which is vital in acute pain, namely to provide the substrate for “fight or flight responses”. Since the medullary triad is a gateway from descending modulation from the brain to the spinal cord, we should study in the future how the deregulation of the RVM-VLM-DRt triad in balancing inhibition and facilitation of pain may account for chronic pain generation and/or maintenance.

## Author Contributions

IT was responsible for the overall organization of the manuscript and wrote the parts related to the rostroventromedial medulla and the ventrolateral medulla. IM was responsible for the items related to the role of the dorsal reticular nucleus. The overall manuscript was reviewed by both authors.

## Conflict of Interest Statement

The authors declare that the research was conducted in the absence of any commercial or financial relationships that could be construed as a potential conflict of interest. The reviewer ES and handling Editor declared their shared affiliation, and the handling Editor states that the process nevertheless met the standards of a fair and objective review.

## Abbreviations

AT_1_: angiotensin type 1 receptors
DNIC: diffuse noxious inhibitory control
DRt: dorsal reticular nucleus
fMRI: functional magnetic resonance imaging
HSV-1: Herpes Simplex virus type 1
LC: locus coeruleus
LRt: lateral reticular nucleus
NRM: nucleus raphe-magnus
NTS: nucleus of the solitary tract
PAG: periaqueductal gray
RF: reticular formation
RVM: rostroventromedial medulla
Sp5C: spinal trigeminal nucleus
TRPV1: vanilloid receptor type 1
VLM: caudal ventrolateral medulla
VLMlat: caudalmost part of the VLM
WDR: wide-dynamic range.
